# Heterogeneidades temporal e espacial da cobertura vacinal infantil nas capitais brasileiras: proposta metodológica utilizando razão de doses aplicadas e esperadas

**DOI:** 10.1590/0102-311XPT075025

**Published:** 2026-02-06

**Authors:** Mateus Henrique Silva Alves, Orozimbo Henriques Campos, Karina Cardoso Meira, Isabel Silva Mitraud Ruas, Taynãna César Simões

**Affiliations:** 1 Universidade Federal de Minas Gerais, Belo Horizonte, Brasil.; 2 Instituto de Educação Continuada, Pontifícia Universidade Católica de Minas Gerais, Contagem, Brasil.; 3 Departamento de Ciências Farmacêuticas, Universidade Federal de São Paulo, São Paulo, Brasil.; 4 Instituto René Rachou, Fundação Oswaldo Cruz, Belo Horizonte, Brasil.

**Keywords:** Cobertura Vacinal, Calendário Básico de Vacinação da Criança, Análise Espaço-Temporal, Vaccination Coverage, Immunization Program, Spatio-Temporal Analysis, Cobertura de Vacunación, Calendario de Inmunizaciones, Análisis Espacio-Temporal

## Abstract

O objetivo foi analisar as heterogeneidades temporal e espacial das coberturas vacinais do calendário básico infantil oferecidas pelo Sistema Único de Saúde (SUS), nas capitais e macrorregiões brasileiras, entre 2010 e 2021, utilizando um novo indicador para monitoramento. Este é um estudo ecológico ao nível das capitais brasileiras e ano de registro das doses. Foi proposto um indicador baseado na razão de doses aplicadas pelo número mínimo esperado de doses para atingir a cobertura vacinal preconizada, no esquema vacinal completo do calendário básico infantil, com as vacinas BCG, hepatite A e B, meningocócica conjugada C e reforço, pentavalente, pneumocócica 10 valente e reforço, poliomielite e reforço, rotavírus humano, tríplice bacteriana, e tríplice viral 1ª e 2ª doses. As razões de doses foram analisadas temporal e espacialmente utilizando modelos aditivos generalizados (GAM, acrônimo em inglês) e modelos autorregressivos condicionais (CAR, acrônimo em inglês). Houve queda significativa das coberturas vacinais para todos os imunobiológicos a partir de 2015, com a maioria das vacinas tendo coberturas abaixo das metas mínimas preconizadas pelo Ministério da Saúde. Observou-se diferenças na magnitude de redução no território brasileiro, sendo maior nas capitais das regiões Norte e Nordeste nos anos mais recentes, em particular, durante a pandemia de COVID-19. O indicador proposto facilitou o tratamento estatístico, utilizando apenas um sistema de informação e as metas preconizadas, possibilitando avaliar quedas vacinais significativas ao longo do tempo, com diferenças entre capitais e macrorregiões. Foram identificadas regiões prioritárias para ações do Programa Nacional de Imunizações (PNI), como intensificação e diversificação de campanhas de imunização, a fim de recuperar as históricas altas coberturas vacinais.

## Abstract (en)

This study aimed to analyze vaccination coverage temporal and spatial heterogeneities in the basic children schedule in the Brazilian Unified National Health System (SUS) in state capitals and macroregions from 2010 to 2021 by a new monitoring indicator. This is an ecological study at the level of Brazilian capitals and year of registration of doses. An indicator based on the ratio between applied doses and the minimum expected number of doses (i.e., the recommended vaccination coverage in the complete basic vaccination schedule for children: BCG, hepatitis A and B, meningococcal conjugate C and its booster, pentavalent, pneumococcal 10-valent and its booster, poliomyelitis and its booster, human rotavirus, triple bacterial, and the first and second triple viral doses) was proposed. Dose ratios were temporally and spatially analyzed by generalized additive and conditional autoregressive models. This study found a significant drop in vaccination coverage for all immunobiologicals from 2015 onward. The coverage of most vaccines remained below the minimum targets of the Brazilian Ministry of Health. This study also observed differences in reduction magnitude (greater in Northern and Northeastern capitals in recent years, particularly during the COVID-19 pandemic). The proposed indicator facilitated statistical treatment, using only an information system and the recommended goals and making it possible to assess significant vaccination drops over time, with differences between capitals and macroregions. This study found priority regions for Brazilian National Immunization Program actions, such as intensifying and diversifying immunization campaigns to achieve the past high vaccination coverage.

## Abstract (es)

El objetivo fue analizar las heterogeneidades temporales y espaciales de la cobertura vacunal del calendario básico infantil ofrecido por el Sistema Único de Salud en las capitales y macrorregiones brasileñas entre 2010 y 2021, utilizando un nuevo indicador para el monitoreo. Se trata de un estudio ecológico a nivel de las capitales brasileñas y del año de registro de las dosis. Se propuso un indicador basado en la relación entre las dosis administradas y el número mínimo esperado de dosis para alcanzar la cobertura vacunal recomendada, en el esquema de vacunación completo del calendario básico infantil, con las vacunas BCG, hepatitis A y B, meningocócica conjugada C y refuerzo, pentavalente, neumocócica 10 valente y refuerzo, poliomielitis y refuerzo, rotavirus humano, triple bacteriana y triple viral 1ª y 2ª dosis. Las razones de las dosis se analizaron temporal y espacialmente utilizando modelos aditivos generalizados (GAM, por su sigla em inglés) y modelos autorregresivos condicionales (CAR, por su sigla em inglés). Desde 2015 se ha producido una caída significativa en la cobertura vacunal de todos los inmunobiológicos, y la mayoría de las vacunas tienen una cobertura inferior a los objetivos mínimos recomendados por el Ministerio de Salud. Se han observado diferencias en la magnitud de la reducción en el territorio brasileño, siendo mayor en las capitales de las regiones Norte y Nordeste en los últimos años, en particular durante la pandemia de COVID-19. El indicador propuesto facilitó el tratamiento estadístico, utilizando solo un sistema de información y las metas recomendadas, lo que permitió evaluar las caídas significativas en la cobertura vacunal a lo largo del tiempo, con diferencias entre capitales y macrorregiones. Se identificaron regiones prioritarias para las acciones del PNI (Programa Nacional de Inmunización), como la intensificación y diversificación de las campañas de inmunización, con el fin de recuperar las altas coberturas vacunales históricas.

## Introdução

O processo de imunização no Brasil é orientado pelo Ministério da Saúde, por meio do Programa Nacional de Imunizações (PNI), e pelas Secretarias Estaduais e Municipais. Essa política pública de saúde de ação tripartite teve relevante papel na redução da mortalidade infantil e aumento da expectativa de vida ao nascer dos brasileiros, com uma redução de 83% das mortes de crianças menores de cinco anos por causas imunopreveníveis em 2017, comparado ao período anterior desde 1996 ^1^. O PNI tem gerado resultados efetivos e, sobretudo, credibilidade junto à população brasileira ao longo dos anos, no entanto, tem-se verificado quedas das coberturas vacinais em anos recentes, o que pode gerar grandes impactos para a saúde pública, resultando no retorno e fortalecimento de algumas doenças de importante morbimortalidade, principalmente em crianças ^2^ ^,^ ^3^.

A vacinação de rotina consiste na execução de um Calendário Nacional de Imunização, que deve ser seguido por cada cidadão a partir de seu nascimento, visando alcançar a prevenção específica das doenças imunopreveníveis nos indivíduos, bem como a indução da imunidade de rebanho, responsável pela interrupção da transmissão coletiva. Para garantir proteção comunitária, um dos fatores necessários é que as coberturas vacinais sejam altas e homogêneas, atingindo de 90 a 100% dos suscetíveis, a depender do imunobiológico ^4^.

A cobertura vacinal é um indicador que estima o nível de proteção da população contra algumas doenças, mediante o cumprimento do esquema completo de imunização, sendo essencial para as análises de situação de saúde e o direcionamento das políticas de saúde da criança ^4^ ^,^ ^5^ ^,^ ^6^. Baixas coberturas vacinais representam risco de acúmulo de suscetíveis, com potencial para introdução e manutenção da circulação de agentes infecciosos ^7^. 

Do ponto de vista quantitativo, o indicador usual, calculado por meio de uma taxa (número de doses/população em risco), apresenta limitações quanto ao tratamento estatístico se comparado a uma proporção com numerador e denominador de mesma natureza, que sejam subconjuntos. Geralmente a cobertura vacinal não apresenta distribuição gaussiana, e são aplicadas transformações algébricas que dificultam a interpretação da medida de associação. Por outro lado, ao se optar por trabalhar com o número de doses aplicadas por meio de um modelo estatístico de probabilidades para dados de contagem que considere a população exposta no preditor linear, é necessária a consulta em pelo menos dois sistemas de informação distintos ^4^ ^,^ ^5^. Frente a essas limitações, neste trabalho é proposto um indicador para analisar a cobertura vacinal a partir de uma razão de doses. Utiliza-se como numerador o número de doses aplicadas no esquema completo e, como denominador, o número mínimo esperado de doses a serem aplicadas à população alvo, a fim de que a cobertura vacinal preconizada pelo Ministério da Saúde seja atingida. Além de utilizar apenas o sistema de informação do PNI, considera-se as metas de cobertura mínima preconizadas para cada vacina neste indicador. 

Com base no indicador proposto e na necessidade de monitoramento contínuo quanto à imunização da população, foram analisadas as heterogeneidades temporal e espacial das coberturas vacinais infantis nas capitais brasileiras, no período de 2010 a 2021, para todos os imunobiológicos do calendário básico infantil. A análise e entendimento da cobertura vacinal permitem subsidiar processos de planejamento, execução, monitoramento e avaliação de políticas públicas relativas à atenção à saúde da criança e ao controle de doenças evitáveis por imunização, além da avaliação operacional e o delineamento de estratégias do PNI ^5^.

## Métodos

Este é um estudo do tipo ecológico com abordagem temporal e espacial das coberturas vacinais ao nível das capitais brasileiras e ano de registro das doses de cada vacina do calendário básico infantil, oferecidas pelo Sistema Único de Saúde (SUS), no período de 2010 a 2021. Fez-se uma proposta metodológica de um indicador para descrição e análise das doses vacinais aplicadas e esperadas sob uma cobertura vacinal mínima preconizada, avaliando a dinâmica no espaço e tempo, baseando-se em dados disponibilizados no Sistema de Informações do Programa Nacional de Imunizações (SI-PNI) do Departamento de Informação e Informática do SUS (DATASUS) ^8^. A busca seguiu as recomendações dos *Guidelines for Accurate and Transparent Health Estimates Reporting: the GATHER Statement* ^9^. 

À princípio, foi feito o download dos dados anuais e segundo capital de notificação no período de 2010 a 2021 no SI-PNI ^8^, seguido da limpeza, organização e harmonização das bases, criando uma base única e integrada. Foram extraídos dados de cobertura e doses aplicadas no esquema completo de cada imunobiológico oferecido pelo SUS, constantes no calendário básico da criança com até 1 ano de idade, além da informação das metas de cobertura vacinal mínimas preconizadas pelo Ministério da Saúde. Os seguintes imunobiológicos foram analisados: BCG (vacina contra formas graves de tuberculose meníngea e miliar), hepatite B, poliomielite e reforço (contra a paralisia infantil), pentavalente (contra difteria, tétano, coqueluche, hepatite B e *Haemophilus influenzae* tipo B), rotavírus humano (contra diarreia), hepatite A, tríplice viral doses D1 e D2 (contra sarampo, caxumba e rubéola), meningocócica conjugada C e reforço (contra meningite meningocócica do tipo C), pneumocócica 10 valente e reforço (contra pneumonia, meningite e otite) e tríplice bacteriana (contra difteria, tétano e coqueluche). As metas de cobertura vacinal mínimas preconizadas pelo Ministério da Saúde para cada imunobiológico são de 100% para a tríplice bacteriana, de 90% para BCG e rotavírus e de 95% para as demais vacinas.

Tipicamente, em um desenho de estudo epidemiológico do tipo ecológico, as informações de interesse são agregadas no tempo e/ou espaço, ou em grupos populacionais específicos. Nas análises de tendência temporal e distribuição espacial, geralmente os modelos inferenciais estatísticos atribuem uma distribuição de probabilidade para variáveis do tipo contagem do evento de interesse, como as distribuições de Poisson ou Binomial Negativa ^10^ ^,^ ^11^. Além disso, na avaliação da ocorrência de um agravo em saúde, é comum analisar uma medida de prevalência ou taxa de incidência, que geralmente, não segue uma distribuição gaussiana. Uma estratégia para se utilizar o modelo de contagem, é desmembrar indicadores do tipo razão, tendo o numerador como a variável resposta na unidade de tempo e/ou espaço *i* (*Y*_*i*_ ), e denominador (*N*_*i*_ ) como o tamanho da população em risco ou exposta ao evento de interesse no tempo e/ou espaço *i*, que é transformado na escala logarítmica natural e adicionado ao preditor linear como um termo denominado *offset* (*ln*(*N*_*i*_ )):

(1) $$Y_{i}\text{∼Poisson}\left(\mu_{i}{\text{=θ}}_{i}N_{i}\right)$$

(2) $$\text{log}\left(\mu_{i}\right){\text{=β}}_{i}X_{i}\text{+ln}\left(N_{i}\right)$$

Após o ajuste do modelo (1), a exponenciação do(s) parâmetro(s) estimado(s) da(s) variável(is) preditora(s), por exemplo, o ano ou área de ocorrência do evento (*X*_*i*_ ), pode ser interpretada como uma medida de associação, tal como risco relativo (RR) ou razão de taxas/prevalências (*exp* (*θ*_*i*_ *= X*_*i*_ *β*_*i*_ )), *i = 1*, ..., *N*, sendo *N* o período total de tempo e/ou unidades espaciais de análise. A distribuição Binomial Negativa é uma extensão do modelo de Poisson, com um parâmetro extra de dispersão dos dados ^10^.

A cobertura vacinal das vacinas do calendário básico infantil pode ser entendida como a proporção de crianças com até um ano de idade que receberam o esquema completo de vacinação, em relação ao total de crianças na mesma faixa etária na população. Por esquema completo, entende-se a aplicação de todas as doses de cada vacina como preconizado pelo PNI, nas faixas etárias e intervalos corretos. O número de doses necessárias e os intervalos recomendados entre as doses para cada tipo de vacina constam de normas nacionais estabelecidas pelo Ministério da Saúde ^4^.

No caso do indicador de cobertura vacinal do SI-PNI, o numerador é o número de doses aplicadas no esquema completo no ano/área *i* (*Y*_*i*_ ) e o denominador, o número de crianças com até um ano de idade ou nascidos vivos no ano/área *i* (*N*_*i*_ ). No entanto, ao exponenciar a estimativa do parâmetro de interesse, tem-se uma medida de difícil interpretação epidemiológica (número de doses/número de crianças), por não se tratar de uma proporção em que o numerador é um subconjunto do denominador.

Neste estudo, ao invés de se utilizar, como termo *offset*, o logaritmo natural da população com até 1 ano de idade ou nascidos vivos no modelo (1), propõe-se utilizar o número mínimo esperado de doses a serem aplicadas à população alvo, para que a cobertura vacinal preconizada seja atingida. Neste estudo, a razão de doses (*RD*_*i*_ ) compara o número de doses efetivamente aplicadas (*D*_*i*_ ) com o número de doses esperadas para se atingir uma cobertura vacinal mínima (*D_esp*_*i*_ ), *i = 1*, ..., *N*:

(3) $${\text{RD}}_{i}=\frac{D_{i}}{{\text{Desp}}_{i}}$$

sendo,

(4) $${\text{Desp}}_{i}=\frac{D_{i}*{\text{Meta}}_{k}}{{\text{CV}}_{i}}$$

Em que *CV*_*i*_ é a cobertura vacinal observada e *Meta*_*k*_ é a cobertura vacinal mínima preconizada, com *k = 1, 2,* 3, a depender do imunobiológico avaliado (metas de 90, 95 e 100%, respectivamente). Neste contexto, no modelo proposto em (1), *Y*_*i*_ *= D*_*i*_ *e N*_*i*_ *= D_esp*_*i*_ . Sendo esta razão significativamente menor que 1 (um), considera-se que o número mínimo de doses aplicadas não foi atingido e que, portanto, a cobertura vacinal está abaixo do preconizado.

Com base no novo indicador, foram utilizadas ferramentas descritivas, métodos temporais e espaciais para analisar a existência de heterogeneidade nas distribuições de cobertura vacinal dos imunobiológicos do calendário infantil nas capitais, também agregadas por macrorregiões brasileiras, no período de 2010 a 2021. As coberturas vacinais foram descritas por medidas-resumo estatísticas e análise gráfica, avaliação da distribuição temporal, além de mapeamento. Denominou-se cobertura vacinal global a cobertura média, considerando todos os imunobiológicos analisados. Foram feitos *boxplots* da cobertura vacinal global anual e gráficos de séries temporais das coberturas vacinais anuais de cada imunobiológico segundo as macrorregiões brasileiras, tabelas com as taxas anuais no início e final das séries temporais, queda percentual e diferença percentual para as metas de cobertura vacinal preconizadas, para todo o país e segundo as macrorregiões.

As coberturas vacinais de cada capital foram descritas em mapas temáticos subdivididos segundo os estados brasileiros, ou seja, a divisão administrativa estadual representa a informação apenas da capital, em que o gradiente de cores varia do cinza claro (mais baixa cobertura vacinal) ao preto (maior cobertura vacinal). Foram criados mapas da cobertura vacinal média global e de cada vacina para os 4 triênios do período (2010-2012, 2013-2015, 2016-2018, 2019-2021). No caso de dados faltantes para determinadas vacinas, a média foi calculada em biênios, de modo a se ajustar aos dados obtidos.

A partir dos indicadores de cobertura vacinal e doses aplicadas, foi calculado o número mínimo de doses esperadas a serem aplicadas, caso a meta do Ministério da Saúde fosse atingida, como proposto na Equação 2. Os momentos do tempo em que a razão tenha sido menor do que 1 e que houvesse mudanças significativas na curva estimada dessas razões (tendências descendentes apontando quedas das coberturas vacinais) foram avaliadas por meio de Modelos Aditivos Generalizados (GAM, acrônimo em inglês), sendo o preditor linear na Equação 1 acrescido de uma função suavizadora ou termo *spline* (*thin plate regression*) no ano de aplicação da dose. Utilizando os modelos GAM com efeito fixo de macrorregião e interação da macrorregião com o tempo no termo suavizado, foram obtidas as estimativas médias de razão de doses por região de residência, tendo a Região Norte a categoria de referência para cada vacina, tendo como resultado, gráficos que permitem a visualização das curvas estimadas de razão de doses no período, para cada macrorregião ^12^. 

Com os resultados dos modelos GAM, foi possível verificar a partir de que ano uma determinada vacina apresentou cobertura vacinal abaixo do esperado, ou seja, quando o número de doses aplicadas foi menor que o número de doses esperadas (razão de doses < 1), considerando a cobertura vacinal mínima preconizada (primeiro ano que a curva atinge valores abaixo da reta horizontal em *y = 1*). 

Posteriormente, as razões de doses estimadas e suavizadas para cada capital brasileira foram obtidas por meio de Modelos Autorregressivos Condicionais (CAR, acrônimo em inglês) ^13^, que foram estimados segundo o método determinístico INLA ^14^, para o último período de observação (2019-2021). Os resultados da modelagem (GAM e CAR) foram apresentados para o imunobiológico com maior queda percentual no período de análise. Todas as análises foram feitas no software estatístico R (versão 4.1.3, http://www.r-project.org), usando os pacotes *mgcv*, INLA, *tidyverse* e *ggplot2*.

Quanto às considerações éticas, a pesquisa foi realizada com dados de acesso livre, em que não há identificação dos sujeitos e, portanto, não houve necessidade de ser submetida ao Comitê de Ética em Pesquisa, de acordo com o art. 1 da *Resolução CNS nº 510*, de 7 de abril de 2016 ^15^ e *Resolução nº 674*, de 6 de maio de 2022 ^16^.

## Resultados

A cobertura vacinal global mediana no período foi de 82,4% (média = 83,6%), com desvio interquartílico de 20% (percentis 25 e 75 de 73% e 93%, respectivamente). As médias globais anuais foram de 75,6% (2010), 97,4% (2011), 87,3% (2012), 89,8% (2013), 87,5% (2014), 91,4% (2015), 90,5% (2016), 80% (2017), 84,8% (2018), 82,9% (2019), 71,2% (2020), e 55,3% (2021). As medianas (quartil 1-quartil 3) no período para cada imunobiológico foram de 112,2% (103,8-125,9) para BCG, 73,4% (68,4-77,8) para hepatite A, 93% (86,6-104,1) para hepatite B, 80,1% (79,7-93,2) para meningocócica C e de 77,1% (72,9-83,2) para o reforço, 73,4% (69,2-87,1) para pentavalente, 78,3% (79,9-86,8) para pneumocócica e 75,4% (70,0-82,3) para o reforço, 84,1% (79,8-93,5) para poliomielite e 70,4% (69,6-77,3) para o reforço, 81% (81,0-86,8) para rotavírus, 90,5% (89,3-91,9) para tríplice bacteriana, e 89,8% (87,3-98,0) e 67,9% (59,8-77,3) para as 1ª e 2ª doses de tríplice viral, respectivamente.

A Tabela 1 mostra a cobertura vacinal observada para cada imunobiológico nos anos inicial (2010) e final (2021) do período considerado, a diferença percentual no período e a diferença percentual na cobertura vacinal do último ano em relação à meta preconizada. Para todo o país, houve maior queda para a BCG no período de 2010 a 2021 (-59,3%), seguido da poliomielite e reforço da tríplice viral 1ª dose (-40,1%). Todas as vacinas ficaram abaixo da cobertura vacinal mínima, verificando-se uma diferença percentual da meta, variando de -56,7% (reforço da tríplice viral 2ª dose) a -9,2% (tríplice bacteriana − DTP) no último ano. Algumas vacinas, embora tenham apresentado aumento da cobertura vacinal no período, como a meningococo C (+39,6%), apresentaram grandes diferenças percentuais abaixo da meta (-38,8%).

**Tabela 1:** Distribuição das coberturas vacinais em 2010 e 2021, diferença percentual no período e diferença percentual da meta de cobertura vacinal mínima preconizada segundo imunobiológico para todo o Brasil.

Imunobiológico	Cobertura 2010	Cobertura 2021	Diferença no período (%)	Diferença da meta (%)
Poliomielite (reforço)	92,9	49,4	-43,5	-45,6
Hepatite B *	88,5	55,5	-33,0	-39,5
Hepatite A *	60,1	55,6	-4,5	-39,4
Poliomielite	88,5	57,4	-31,1	-37,6
Pentavalente *	24,9	57,8	+32,9	-37,2
Meningocócica C	26,9	58,6	+31,7	-36,4
Tríplice viral	99,9	61,4	-38,5	-33,6
Rotavírus	83,0	58,4	-24,6	-31,6
BCG	107,0	60,7	-46,3	-29,3
Tríplice bacteriana (reforço) *	98,0	89,5	-8,5	-10,5

* Dados incompletos para o período: hepatite A e B − 2014 a 2021; pentavalente − 2012 a 2021; reforço DTP − 2010 a 2016.

Considerando as macrorregiões, a Tabela 2 apresenta as quedas percentuais no período e a diferença da cobertura vacinal no final do período para as metas. É possível perceber que, em todas as macrorregiões, houve queda das coberturas vacinais no período para os imunobiológicos BCG, hepatite B, meningococo C reforço, pneumocócica 10 reforço, poliomielite e o reforço, rotavírus, e tríplice viral 2ª dose. Em todas as regiões, as coberturas vacinais ficaram abaixo das metas preconizadas para todas as vacinas no último ano de análise. Deve-se destacar os baixos percentuais abaixo das metas da tríplice viral 2ª dose nas capitais das regiões Norte, Nordeste, Centro-oeste e Sudeste, do reforço da poliomielite no Sudeste e hepatite B no Sul.

**Tabela 2:** Distribuição das coberturas vacinais em 2010 e 2021, percentual de queda no período e diferença da meta preconizada, nas macrorregiões, segundo imunobiológico para todo o Brasil.

Imunobiológico	Norte (diferença)	Nordeste (diferença)	Centro-oeste (diferença)	Sudeste (diferença)	Sul (diferença)
BCG	40,5 (10,8)	75,1 (29,5)	49,1 (28,6)	60,7 (19,4)	67,4 (40,8)
Hepatite B **	11,7 (12,2)	64,0 (30,2)	44,9 (29,4)	16,8 (28,5)	4,1 (39,5)
Poliomielite	42,8 (37,7)	45,4 (41,6)	34,9 (30,1)	39,8 (32,1)	25,7 (28,2)
Pentavalente **	-27,8 (37,7)	-26,5 (41,0)	-29,6 (29,9)	-24,5 (32,1)	-34,2 (28,0)
Rotavírus	21,2 (35,3)	24,6 (39,2)	23,1 (27,6)	26,4 (31,3)	21,2 (25,5)
Meningocócica C	-51,9 (34,5)	-35,0 (39,3)	-48,9 (27,7)	-9,5 (31,5)	-52,5 (27,0)
Hepatite A	-6,1 (40,2)	19,1 (42,4)	15,4 (30,2)	-3,9 (26,5)	14,1 (26,0)
Tríplice viral	33,1 (30,5)	54,6 (37,0)	25,8 (22,9)	31,3 (27,9)	27,1 (25,5)
Tríplice bacteriana	4,9 (1,1)	6,8 (3,5)	-5,3 (-11,2)	-4,6 (-6,0)	1,5 (2,7)
Polio reforço	33,4 (45,4)	44,4 (48,7)	37,8 (34,0)	37,2 (38,8)	18,5 (35,2)

* Diferença da meta preconizada.

** Dados incompletos para o período: hepatite A e B − 2014 a 2021; pentavalente − 2012 a 2021.

**Figura 1:**
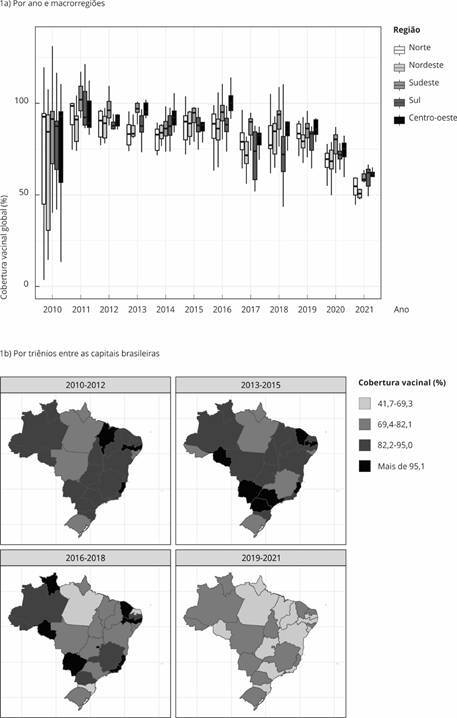
Distribuições temporais das coberturas vacinais globais considerando todos os imunobiológicos: por ano e macrorregiões, e por triênios entre as capitais brasileiras. Brasil, 2010-2021.

A Figura 1a mostra simultaneamente as tendências de decrescimento das coberturas vacinais global e por macrorregiões brasileiras, além das disparidades de cobertura vacinal globais anuais entre as macrorregiões, destacando-se as menores coberturas vacinais nas capitais das regiões Norte e Nordeste, e com maior desigualdade nos anos da pandemia de COVID-19 (2020-2021). A Figura 1b apresenta a distribuição espacial das coberturas vacinais globais entre as capitais brasileiras, representadas por seus respectivos estados no mapa. Quanto mais clara a cor da escala nos mapas, menor a cobertura vacinal. Nos quatro triênios do período, é possível verificar a queda das coberturas em todo o território. Essas coberturas ficaram abaixo das metas preconizadas em todas as capitais no último triênio.

**Figura 2:**
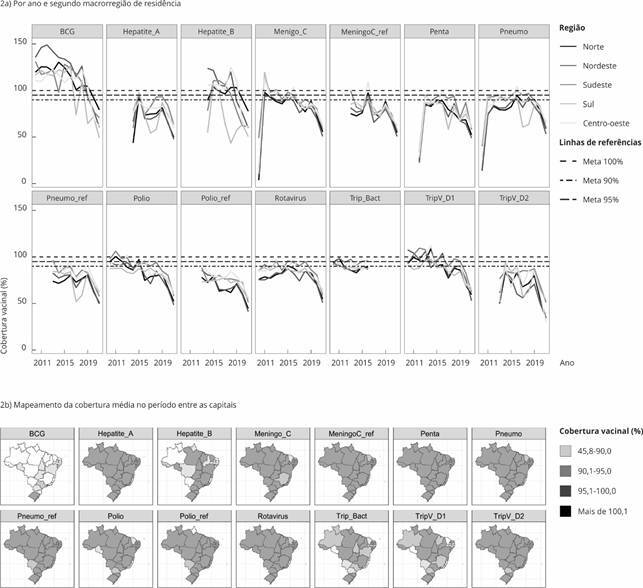
Distribuição temporal das coberturas vacinais segundo o tipo de vacina, em cada ano e segundo a macrorregião de residência, e mapeamento da cobertura média no período entre as capitais. Brasil, 2010-2021.

BCG: tuberculose meníngea e miliar; Hepatite_A: hepatite A; Hepatite_B: hepatite B; Meningo_C: meningocócica C; MeningoC_ref: meningocócica C reforço; Penta: pentavalente; Pneumo: pneumocócica; Pneumo_ref: pneumocócica reforço; Polio: poliomielite; Polio_ref: poliomielite reforço; Rotavírus: rotavírus; Trip_Bact: tríplice bacteriana; TripV_D1: tríplice vira D1; TripV_D2: tríplice viral D2.

Na Figura 2a, são mostradas as diferenças nas distribuições temporais de cobertura vacinal de cada vacina, segundo a macrorregião de residência. As linhas pontilhadas horizontais representam as metas preconizadas de cobertura vacinal mínima de 90%, 95% e 100%. Observa-se a ausência de dados para alguns imunobiológicos e verifica-se, de forma mais clara e específica, a queda das coberturas vacinais para todos os imunobiológicos, que em alguns casos se apresentam abaixo da meta em todo o período analisado, a exemplo do reforço da pneumocócica (Pneumo_ref), hepatite A e tríplice viral D2. A Figura 2b, mostra a distribuição espacial da cobertura vacinal média no período de cada imunobiológico nas capitais brasileiras, representadas no mapa por seus respectivos estados. Grande parte dos imunobiológicos apresentam dispersão espacial de baixas coberturas em todas as capitais no período, tais como pentavalente (Penta), hepatite A, meningocócica C reforço (MeningoC_ref), pneumocócica reforço (Pneumo_ref), poliomielite reforço (Polio_ref), e tríplice viral D2 (TripV_D2). 

**Figura 3:**
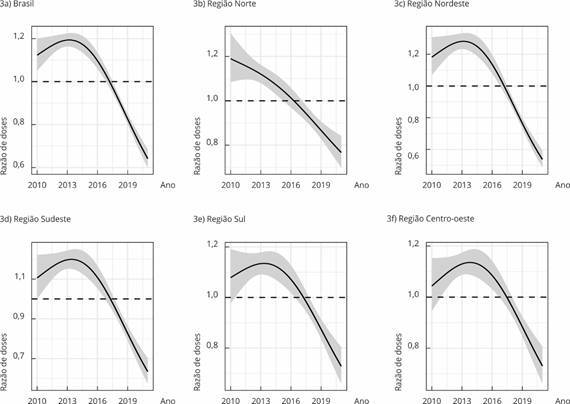
Curvas estimadas da razão de doses segundo Modelo Aditivo Generalizado (GAM, acrônimo em inglês) para a razão de doses da vacina BCG segundo macrorregião. Brasil, 2010 a 2021.

A fim de avaliar a significância estatística do número de doses insuficientes e de queda na cobertura, foram ajustados os modelos GAM para cada tipo de vacina. A distribuição Binomial Negativa apresentou o melhor ajuste. Como exemplo, observa-se a curva suavizada estimada da razão de doses para a BCG e os resultados para as demais vacinas são descritos no texto. Em todas as macrorregiões, a razão de doses foi menor que 1 (um), ou seja, cobertura vacinal menor que a preconizada, a partir de 2018, apresentando tendência decrescente desde 2014 (Figura 3). Embora tenha havido queda da cobertura vacinal em todas as regiões, houve diferenças em relação ao momento do tempo dessa evidência. Houve queda a partir de 2014 em todas as regiões, exceto na Norte (queda em 2010). Não houve diferença da razão de doses entre as regiões Norte e Nordeste, que são menores que aquelas nas regiões Centro-Sul. As coberturas estiveram abaixo do esperado a partir de 2017 no Norte, 2018 no Nordeste, Sudeste e Sul, e a partir de 2019 no Centro-oeste. 

Dentre as demais evidências obtidas com os modelos GAM, verificou-se queda da cobertura vacinal, a partir de 2018, para poliomielite e tríplice viral D1; em 2019 para hepatite B, meningocócica C reforço, pneumocócica C reforço, poliomielite reforço, rotavírus, e em 2020 para tríplice viral D2. Todas as vacinas (exceto tríplice bacteriana, que não contém dados para o período inteiro) apresentaram tendência de queda da cobertura vacinal nos últimos anos. Essa queda se iniciou em 2010 para os reforços de meningocócica e poliomielite, em 2012 para BCG, em 2015 para poliomielite e tríplice viral D1, em 2016 para reforço de pneumocócica e rotavírus, em 2017 para hepatite B, meningocócica C, pneumocócica e tríplice viral (D2), e em 2018 para pentavalente e hepatite A (dados não mostrados).

Quanto ao efeito fixo da macrorregião, não houve diferença das regiões em relação à Norte (macrorregião de referência) para a vacina da hepatite A. Contudo, houve tendência de queda desde 2017, ficando a cobertura abaixo do esperado entre 2017 e 2019 para o Norte, e, a partir de 2020, para o Nordeste. Para hepatite B, as regiões Sul e Sudeste apresentaram coberturas vacinais maiores que a Região Norte. Houve tendência de queda na cobertura a partir de 2016, estando abaixo do esperado no Sudeste a partir de 2018, e nas regiões Nordeste e Centro-oeste a partir de 2020. Para a meningocócica C, as regiões não diferiram e houve tendência de queda a partir de 2016, porém sem coberturas abaixo da meta. Para a dose de reforço também não houve diferença das regiões comparadas à Região Norte. Houve tendência de queda a partir de 2017, com cobertura abaixo do esperado a partir de 2017 no Sudeste, e, a partir de 2019, no Nordeste e Centro-oeste. 

Para a pentavalente e pneumocócica, também não houve diferença entre as regiões. Houve tendência de queda para ambas as vacinas a partir de 2017. Para pentavalente, houve cobertura vacinal abaixo do esperado nas regiões Norte e Nordeste nos anos 2017 e 2018. Para a pneumocócica, a cobertura vacinal foi baixa nas regiões Norte e Centro-oeste entre 2016 e 2019, e no Sudeste entre 2016 e 2018. Para o reforço de pneumocócica, houve tendência de queda a partir de 2016 no Norte, Nordeste e Sudeste, e em 2010 no Sul e Centro-oeste. A cobertura vacinal esteve abaixo do esperado nas regiões Sudeste e Centro-oeste a partir de 2017, e no Nordeste a partir de 2016.

Para poliomielite, a única região que difere da Norte é a Sudeste, que possui a melhor cobertura e onde houve tendência de queda a partir de 2014. A Região Norte teve queda contínua, e as demais regiões, quedas desde 2014-2015. Houve baixa cobertura vacinal a partir de 2019 no Sul, e de 2018 para as demais regiões. Para o reforço da poliomielite, a Região Norte se assemelha à Nordeste, com as menores coberturas. Houve queda contínua em todas as regiões, exceto no Sul que começou em 2016. 

A cobertura vacinal de rotavírus apresentou tendência de queda desde 2015, estando abaixo do esperado a partir de 2019 para o Sudeste e Centro-oeste, e, a partir de 2020, para o Norte. Para a tríplice bacteriana, as regiões foram semelhantes. Houve tendência de aumento na cobertura vacinal para todas as regiões no fim do período, exceto no Sul. No entanto, para esse caso é necessário atentar-se ao período dos dados, que termina em 2016. Para a 2ª dose da tríplice viral, a Região Norte se assemelha fortemente à Região Nordeste, e difere das regiões Sul e Sudeste com melhores coberturas vacinais. As tendências de queda no período ocorreram a partir de 2016-2017. Houve cobertura vacinal abaixo do esperado a partir de 2019 para o Nordeste e Centro-oeste, e de 2020 para o Norte e Sudeste. A tríplice viral não apresentou diferença significativa entre as regiões comparadas à Norte. As tendências de queda começaram em 2014, exceto na Região Norte, que apresentou queda consistente em todo o período, desde 2010. A cobertura vacinal ficou abaixo do esperado a partir de 2017 na Região Norte, de 2018 para o Nordeste, e de 2019 para as demais regiões.

**Figura 4:**
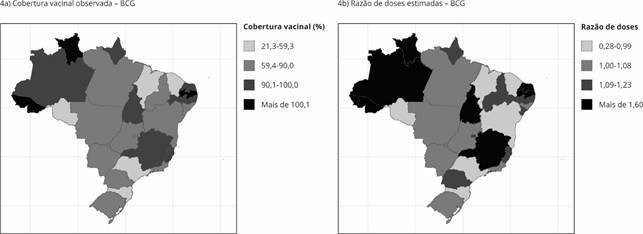
Distribuições espaciais das coberturas vacinais observadas e das razões de doses estimadas pelo Modelo Autorregressivo Condicional (CAR, acrônimo em inglês), para a vacina BCG nas capitais brasileiras, no triênio 2019-2021.

Somado às diferenças ao longo do tempo, houve heterogeneidade espacial e temporal das coberturas de todos os imunobiológicos do calendário básico infantil entre as capitais brasileiras, a exemplo da cobertura vacinal para BCG no triênio 2019-2021 (Figura 4a), que apresenta várias capitais com cobertura vacinal abaixo do preconizado. O modelo CAR estima as razões de doses entre as capitais e evidencia cobertura vacinal da BCG significativamente abaixo do esperado (P(razão de doses < 1) > 80%) no triênio 2019-2021 para as capitais Porto Velho (Rondônia), Florianópolis (Santa Catarina), São Paulo, Salvador (Bahia), São Luís (Maranhão) e Fortaleza (Ceará) (Figura 4b). Padrões semelhantes de quedas foram observados para os demais imunobiológicos.

## Discussão

O PNI constitui uma das principais políticas públicas do sistema de saúde brasileiro. Historicamente, demonstrou elevada efetividade, promovendo redução substancial na incidência e eliminação de algumas doenças, tais como sarampo, poliomielite e tétano neonatal, além da eliminação da rubéola e da síndrome da rubéola congênita. Segundo Domingues et al. ^1^, o sucesso do PNI pode ser atribuído à sua aderência aos princípios fundamentais do SUS de universalidade, equidade, e ao princípio organizativo de descentralização. Na avaliação do PNI, o indicador de saúde de cobertura vacinal é reconhecido como um instrumento essencial para subsidiar o processo de tomada de decisão nas distintas esferas de gestão em saúde pública ^13^.

No entanto, mesmo com a atuação do PNI, nos últimos anos tem-se observado uma diminuição significativa nas cobertura vacinal do calendário básico infantil no Brasil. Diante desse panorama, este estudo utilizou um indicador proposto para analisar as heterogeneidades temporal e espacial das coberturas vacinais, baseado nas metas recomendadas. O indicador mantém a essência da avaliação do indicador de cobertura vacinal usual (número de doses/número de crianças), contudo, oferece vantagens adicionais em termos de análise estatística e interpretabilidade epidemiológica, por se basear em uma proporção (razão de doses aplicadas no esquema completo/o número mínimo esperado de doses a serem aplicadas à população alvo, para que a cobertura vacinal preconizada seja atingida), além de considerar intrinsecamente as metas mínimas preconizadas pelo Ministério da Saúde, e utilizar apenas um sistema de informação evitando possíveis incongruências. Adaptação análoga pode ser pensada para indicadores que se baseiam na cobertura vacinal, tal como a homogeneidade de cobertura vacinal.

Este estudo revelou tendência de decrescimento e disparidades significativas das coberturas vacinais dos imunobiológicos do calendário infantil entre as capitais e macrorregiões do Brasil entre 2010 e 2021. Apesar do grande impacto na diminuição das cobertura vacinal no período da pandemia de COVID-19, observou-se uma tendência geral de queda já nos períodos anteriores, com maior força, a partir de 2016, para a maioria das vacinas, mas com variações regionais e temporais, especialmente nas regiões Norte e Nordeste. Nestas regiões, as reduções de cobertura vacinal ocorreram em diferentes momentos, com maior impacto nos anos mais recentes (2020-2021), e uma dispersão espacial de baixas coberturas foi destacada para várias vacinas, a exemplo da pentavalente (Penta), hepatite A, os reforços de meningocócica C, pneumocócica e poliomielite, além da segunda dose da tríplice viral. Chama atenção a queda substancial observada desde 2010 para BCG e tríplice viral na Região Norte.

As coberturas vacinais e a homogeneidade dos imunobiológicos constantes no calendário básico infantil vêm apresentando quedas significativas desde 2016, após anos de sucesso em alcançar as metas preconizadas, sendo que as vacinas com mais de uma dose perdem cobertura progressivamente ^17^. Um estudo ecológico avaliou o impacto da pandemia de COVID-19 na vacinação de crianças de até um ano de idade. As coberturas vacinais de 10 vacinas presentes no calendário do PNI, no período de 2013 a 2020, foram avaliadas. O ano de 2020 registrou o menor valor de cobertura vacinal (75,1%) considerando a média de todas as vacinas estudadas, enquanto, em 2019, a média ficou em 84,4%. Além disso, das 10 vacinas analisadas, 9 registraram seu menor valor considerando a série histórica, todas com valores muito abaixo das metas ^18^.

Outros estudos têm evidenciado menores coberturas vacinais, maiores taxas de abandono e menores homogeneidades de cobertura vacinal nos estados e municípios das regiões Norte e Nordeste ^18^ ^,^ ^19^ ^,^ ^20^ ^,^ ^21^ ^,^ ^22^. No período de 2006 a 2016, os estados do Pará, Maranhão e Bahia mostraram declínio mais acelerado nas coberturas vacinais da BCG, poliomielite e tríplice viral do que o restante do país. Outros estados do Norte e Nordeste igualmente apresentaram reduções significativas nas coberturas vacinais, como Piauí, Tocantins, Acre, Rondônia, Amapá e Amazonas ^22^. As Unidades Federativas dessas regiões também foram responsáveis pelas maiores taxas de abandono da 3ª dose da vacina pentavalente entre os anos de 2015 e 2016 ^21^. As regiões Norte e Nordeste apresentam desigualdades socioeconômicas e iniquidades no acesso aos serviços de saúde e foram intensamente afetadas pela pandemia de COVID-19, que, além das altas taxas de incidência e mortalidade, promoveu elevadas taxas de desemprego e alta prevalência de insegurança ^23^ ^,^ ^24^ ^,^ ^25^ ^,^ ^26^. Tal conjuntura pode ter contribuído para o pior cenário de baixas coberturas vacinais nos primeiros dois anos da pandemia.

O início das quedas nas coberturas vacinais foi heterogêneo a depender do imunobiológico. Neste estudo, o Brasil apresentou queda significativa em 2010 para os reforços de meningocócica e poliomielite, em 2012 para BCG, em 2015 para poliomielite e tríplice viral D1, em 2016 para reforço de pneumocócica e rotavírus, em 2017 para hepatite B, meningocócica C, pneumocócica e tríplice viral D2, e em 2018 para pentavalente e hepatite A. A queda iniciada em diferentes momentos para cada vacina reflete falhas progressivas no sistema de saúde. Essas mudanças ao longo do tempo destacam a necessidade de vigilância contínua e ajustes rápidos no PNI. Esse cenário é preocupante, pois pode aumentar o risco de reaparecimento de doenças anteriormente controladas ou erradicadas, o que pode levar a surtos epidêmicos, especialmente em territórios com alta vulnerabilidade socioeconômica e em crianças em situação de insegurança alimentar e más condições de moradia ^18^ ^,^ ^19^ ^,^ ^20^ ^,^ ^21^ ^,^ ^22^.

A redução da cobertura vacinal é um problema de saúde pública mundial multideterminado. Entre os possíveis fatores, citam-se a hesitação vacinal, em consequência da falta de consciência sobre a importância da vacinação, da disseminação de notícias falsas, especialmente nas redes sociais, sobre os supostos malefícios das vacinas, entre outros fatores ^27^ ^,^ ^28^ ^,^ ^29^. Paradoxalmente, apesar das numerosas conquistas alcançadas, o PNI enfrenta grandes desafios. Muitas doenças que foram controladas ou erradicadas tornaram-se desconhecidas para a população, levando algumas pessoas a subestimarem a gravidade que essas doenças representam ^1^ ^,^ ^29^. Somado a esses fatores, há o movimento antivacinas que minimiza os benefícios da vacinação e maximiza os riscos, gerando o fenômeno da hesitação vacinal (adiamento na implementação do esquema vacinal ou a recusa em receber vacinas recomendadas, mesmo quando estão disponíveis nos serviços de saúde) ^29^ ^,^ ^30^. Além disso, há a limitação de acesso às vacinas, como resultado de horários reduzidos de atendimento nos postos de saúde, falta de insumos, dentre outros fatores ^29^.

Este estudo avalia todas as vacinas do calendário básico infantil que são oferecidas pelo SUS. Além disso, o indicador proposto apresenta maior adequação estatística e interpretabilidade epidemiológica, pois acomoda melhor uma distribuição de probabilidades conhecida, sem a necessidade de se utilizar transformações para tornar a cobertura vacinal aproximadamente normal; utiliza um único sistema de informação, o SI-PNI, sendo desnecessária a busca da população exposta com base em dados censitários e estimativas populacionais, ou na informação de nascidos vivos em outros sistemas de informação; além de incorporar em seu cálculo, as metas de cobertura vacinal mínima preconizadas pelo Ministério da Saúde. 

Sabe-se que os indicadores utilizados possuem uma série de limitações. O cálculo da cobertura vacinal e, portanto, da razão de doses, está sujeito a erros, tais como a contabilização do número de frascos de vacina utilizados, tomados como estimativas das doses aplicadas, e a incorreção na identificação das doses realmente aplicadas aos menores de 1 ano. A qualidade dos dados administrativos parece ser afetada pelo tipo de vacina, havendo imprecisões do registro de doses aplicadas, principalmente durante a realização de campanhas de vacinação. No entanto, resultados do *Inquérito Nacional de Cobertura Vacinal* de 2020, não mostraram grandes diferenças entre os dados das cadernetas infantis e informações administrativas, quando elas são registradas no sistema do PNI. Comparando-se as coberturas vacinais entre as duas fontes, houve cerca de 32% de não registro no sistema informatizado de dados constantes na caderneta e 8% de discordância entre os dados restantes ^29^. 

Além disso, a demanda da população não residente aos postos de vacinação, principalmente em campanhas, dificulta a avaliação da cobertura vacinal. Outra questão é determinar que parcela da população utiliza os serviços públicos de vacinação, sempre que os dados do numerador se refiram às vacinas aplicadas pelos serviços públicos. A qualidade dos dados de sistemas de informação pode ser afetada tanto pelo tipo de vacina considerada, quanto por características próprias do município, tais como tamanho populacional e nível socioeconômico ^4^.

Embora os dados apresentem fragilidades que possam impactar nos indicadores, para fins de vigilância epidemiológica e promoção à saúde das crianças, monitorar as coberturas vacinais ao longo do tempo, bem como identificar particularidades do território é fundamental para o SUS e para a saúde pública. A análise espaço-temporal das coberturas vacinais, em particular, pode dar base para ações diferenciadas do PNI, priorizando regiões de menor cobertura no território brasileiro, e campanhas focadas em imunobiológicos com quedas mais significativas. Finalmente, o presente estudo contribui para a vigilância da cobertura vacinal ao traçar um panorama de dez vacinas do calendário vacinal infantil, e ao propor um novo indicador de cobertura vacinal, que além de apresentar um melhor tratamento estatístico e epidemiológico, compara doses aplicadas com doses esperadas, utilizando-se apenas um sistema de informação, e considerando uma cobertura vacinal mínima preconizada pelo Ministério da Saúde.

## Data Availability

As fontes de informação utilizadas no estudo estão indicadas no corpo do artigo.
